# An Association Between Autoimmune Hepatitis (AIH) and Immunoglobulin G4-Related Disease (IgG4-RD) in a Male Diagnosed With Primary Sjögren’s Syndrome (SJS): A Case-Based Review

**DOI:** 10.7759/cureus.100486

**Published:** 2025-12-31

**Authors:** Raja A Bakhsh, Khaled S Dairi, Ayman Alsebaey, Jouvany Naguib, Hassan Alsolami, Rana S AL-Zaidi, Haneen Khouja, Bashaer S Khawandanah, Turki M Alsulaimani, Mohammad S Malki, Noor M Bin Mahfooz

**Affiliations:** 1 Internal Medicine, King Faisal Specialist Hospital, Makkah, SAU; 2 Internal Medicine, King Faisal Hospital, Makkah, SAU; 3 Hepatology & Gastroenterology, National Liver Institute Menoufia University, Makkah, SAU; 4 Internal Medicine, Assiut University, Assiut, EGY; 5 Gastroenterology, King Faisal Hospital, Makkah, SAU; 6 Laboratory and Blood Bank, Anatomic Pathology Section, King Faisal Hospital, Makkah, SAU; 7 Intensive Care Unit, Ministry of Health Holdings- Althaghor Hospital, Jeddah, SAU; 8 Radiology, Maternity and Children Hospital (MCH), Makkah, SAU; 9 Main Laboratory, King Faisal Hospital, Makkah, SAU

**Keywords:** autoimmune hepatitis (aih), case-based review, immunoglobulin g4-related disease, overlapping autoimmune disorders, primary sjögren’s syndrome (pss)

## Abstract

IgG4-associated disorders (IgG4-RD) are disease states that are marked by a fibrotic inflammatory disease process that can affect hepatic tissue, salivary glands, and other organs in the body. The hepatic expression of this condition, termed IgG4-associated autoimmune hepatitis (IgG4-AIH), occurs infrequently and can resemble conventional autoimmune hepatitis (AIH), especially during concurrent manifestation with primary Sjögren’s syndrome (pSS), thereby creating diagnostic and therapeutic challenges. We report a clinical case involving a 56-year-old gentleman with an established medical background of type 2 diabetes and hypothyroidism who developed right upper quadrant discomfort, jaundice, and laboratory evidence of cholestasis. Workup revealed hypergammaglobulinemia, positive ANA and anti-Ro antibodies, and markedly elevated serum IgG4 (4.31 g/L). Imaging showed hepatomegaly and bilateral parotid gland enlargement. Liver biopsy showed interface hepatitis, rosette formation, storiform fibrosis, and the dense infiltration of IgG4+ plasma cells (7-10/HPF, IgG4:IgG ratio 50-60%). The patient satisfied diagnostic criteria for both IgG4-AIH and pSS. Treatment with prednisolone, hydroxychloroquine, and azathioprine brought about clinical and biochemical improvement. Rituximab was contemplated for escalated therapy due to the overlapping autoimmune conditions and systemic nature of IgG4-RD. This case highlights the diagnostic difficulty of IgG4-AIH when occurring alongside pSS and illustrates the need for liver biopsy with IgG4 immunostaining in testing atypical hepatitis cases. It is essential to differentiate IgG4-AIH from classic AIH or pSS-related liver disease, as it may have a different treatment response and prognosis. Further studies would assist in refining the diagnostic criteria and therapeutic strategies for overlapping autoimmune syndromes.

## Introduction

IgG4 antibody-related disorder represents a multisystem autoimmune fibroinflammatory condition that emerged following its initial identification in 2001 [1], predominantly within autoimmune pancreatitis contexts. Currently, it is recognized with the potential to involve numerous organs, encompassing the pancreas, salivary glands, retroperitoneum, lungs, and hepatobiliary system [2]. Histopathological changes that are typical of IgG4-RD include extensive lymphoplasma cell infiltration with storiform fibrosis, obliterative phlebitis, and elevated levels of IgG4 in the serum [3]. The hepatic manifestations of IgG4-RD are divided into three different phenotypes, including IgG4-related hepatopathy, IgG4-related autoimmune hepatitis (IgG4-AIH), and hepatic inflammatory pseudotumors [2]. Within these variants, IgG4-AIH represents an uncommon yet well-documented clinical entity demonstrating features clinically and histologically resembling classical autoimmune hepatitis while exhibiting substantial infiltration by IgG4+ plasma cells and notably increased concentrations of serum IgG4 [4]. The diagnosis of IgG4-AIH can be particularly challenging, especially when coexisting with other autoimmune diseases, as overlapping serological and clinical features may obscure the histopathologic signature of IgG4-RD. This level of complexity is even increased when dealing with patients who have primary Sjogren syndrome (pSS), an exocrinopathy that shares symptoms such as glandular enlargement, sicca features, and ANA/anti-Ro positivity with IgG4-related sialadenitis, despite having distinct immunopathology [5]. In this case report, we describe a unique and diagnostically complex case of IgG4-related autoimmune hepatitis that occurred in a middle-aged man coexisting with primary Sjogren syndrome. Through this case, we aim to review and synthesize recent literature to highlight the diagnostic approach, histological features, and therapeutic implications of this overlap.

## Case presentation

Patient demographics and history

A 56-year-old gentleman, a chronic smoker, with an established medical background of type 2 diabetes mellitus, hypertension, iatrogenic hypothyroidism (post-radioactive iodine treatment), eczema, and previously treated pulmonary tuberculosis (>20 years ago) as a past history, with no family history of medical concern, presented to the hepatology clinic for evaluation.

Clinical presentation

He has a history of 4 months of dull, non-food-related right upper quadrant abdominal pain and progressive jaundice, nausea, and constipation. He also noted sicca symptoms, including dry mouth and dry eyes, but denied any history of oral/genital ulcers, joint pain, morning stiffness, muscle weakness, or neurological deficits (e.g., foot or hand drop).

Laboratory findings

The patient’s remaining laboratory values were within normal limits and included the following: coagulation parameters (PTT, PT, INR), basic metabolic profile (sodium, potassium, chloride, BUN, creatinine), hemoglobin and hematocrit levels, red cell indices (MCV, MCH), platelet count, serum albumin, and total protein. Immunoglobulin A (IgA), IgM, and total IgG levels (excluding IgG4) were also within reference ranges, along with tumor markers like AFP and pancreatic enzymes (amylase and lipase). Complement components C3 and C4, rheumatoid factor (RF), and serum protein electrophoresis (SPEP) were normal. The viral hepatitis panel (HBsAg, anti-HCV, anti-HBc IgG) and autoantibodies such as anti-dsDNA, anti-Sm, anti-RNP, AMA, and ANCA were all negative.

Table 1 summarizes the patient's abnormal key laboratory results, immunological profile, and serological markers. These findings played a vital role in guiding the diagnostic evaluation and establishing comorbidity between IgG4-related disease and autoimmune hepatitis, as well as primary Sjogren syndrome. 

**Table 1:** Key Abnormal Laboratory Markers This table summarizes the patient’s significant laboratory findings categorized by organ system and test type. It displays the patient's results alongside standard reference ranges and provides a clinical interpretation for each value, highlighting marked elevations in liver enzymes, IgG4 levels, and specific autoimmune antibodies. ALT: alanine aminotransferase; ALP: alkaline phosphatase; ANA: antinuclear antibody; ASMA: anti-smooth muscle antibody; AST: aspartate aminotransferase; ESR: erythrocyte sedimentation rate; fT4: free thyroxine; HbA1c: hemoglobin A1c; IF: immunofluorescence; IgG4: immunoglobulin G4; SSA: Sjögren’s-Syndrome-related antigen A; TSH: thyroid-stimulating hormone; WBCs: white blood cells

Category	Test	Result	Reference Range	Interpretation
Liver Function	ALT	95 U/L	7–56 U/L	Elevated
	AST	97 U/L	5–40 U/L	Elevated
	ALP	281 U/L	44–147 U/L	Elevated
	Total Bilirubin	14.9 µmol/L	0.1–1.2 mg/dL	Elevated
Immunoglobulins	IgG4	4.310 g/L	< 2.01 g/L	Markedly Elevated
	IgG4:IgG ratio	~50–60%	< 40%	Elevated
Hematology	WBCs	18.75 × 10³/µL	4–11 × 10³/µL	Elevated
	ESR	28 mm/h	0–20 mm/h	Mildly Elevated
Thyroid Function	TSH	23.8 mIU/L	0.4–4.0 mIU/L	Elevated
	fT4 (first value)	2.54 ng/dL	0.8–1.8 ng/dL	Elevated
Diabetes Control	HbA1c	7.95%	< 6.5%	Poor Glycemic Control
Autoantibodies	ANA (IF, 1:80, speckled)	Positive	Negative	Positive autoimmune marker
	Anti-Ro (SSA)	31.2 CU	Negative	Positive (supports Sjögren’s)
	ASMA	1:40	Negative	Weakly Positive
Urine Protein	Protein/Creatinine Ratio	6.06	< 0.2	Markedly Elevated (Proteinuria)

Imaging and pathology

CT Abdomen with Contrast (Pancreatic Protocol) showed a normal appearance of the pancreas with no gross focal lesions seen. The liver is mildly enlarged, measuring 19.5 cm in the craniocaudal span, with mild inhomogeneity related to the history of cholestatic liver disease. No intra- or extrahepatic biliary tree dilatation. The gallbladder appears within normal limits. The spleen, both adrenals, and both kidneys, as well as the pelvic organs, appear within normal limits, and there is no gross bowel abnormality. Also, a small indirect inguinal hernia on the left side was noted, containing fat. No free fluid or enlarged lymph nodes were seen in the abdomen or pelvis. The other finding was spondylosis of the spine.

**Figure 1:**
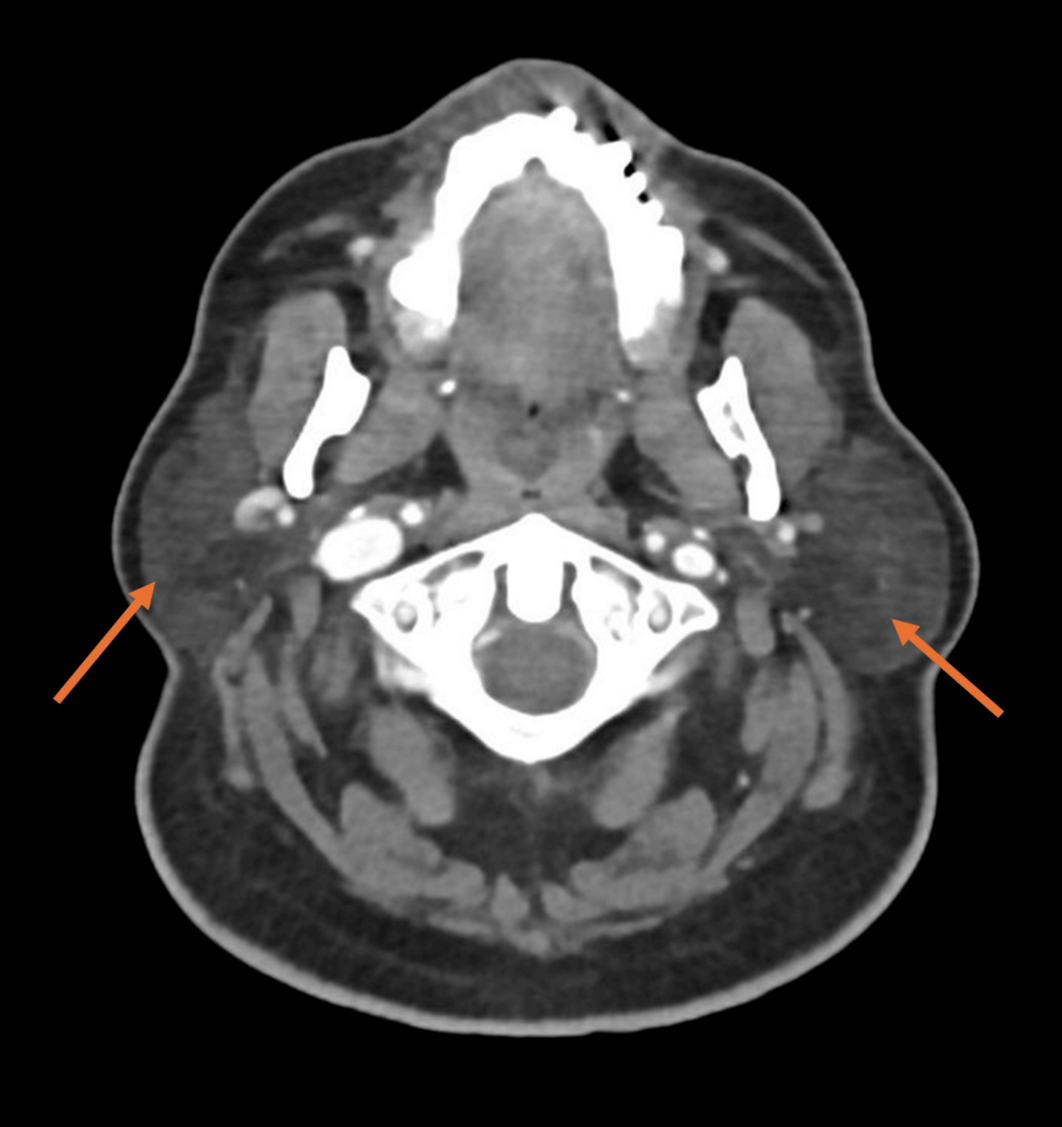
Coronal CT scan of the head shows bilateral parotid gland enlargement.

**Figure 2:**
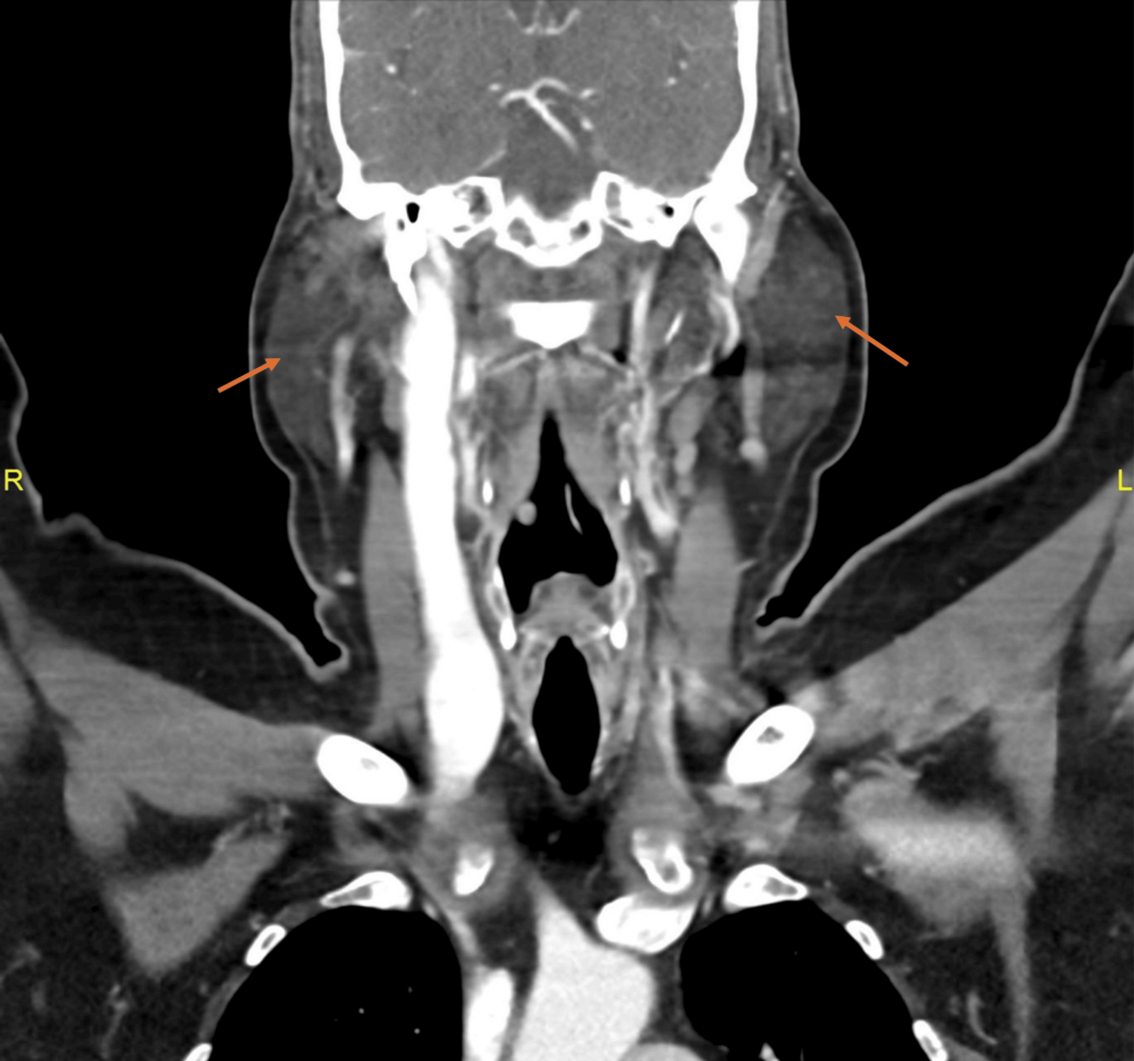
Sagittal CT scan of the head shows bilateral parotid gland enlargement.

CT of the head with contrast showed that the parotid glands are prominent in size bilaterally. The right parotid was 5.5 × 2.3 × 6.2 cm, while the left was 4.5 × 3.0 × 6.2 cm, as shown in Figures 1-2. No definite mass was seen on either side. A few sub-centimetric lymph nodes are seen around the right parotid, especially at the posterior aspect. The largest lymph node was 0.6 × 0.3 cm. A few scattered subcentimetric lymph nodes are also seen in the neck, but no significantly enlarged lymph node is noted. Normal airways of the neck. Normal CT texture of the lung parenchyma with no focal or diffuse abnormal densities.

Normal caliber and distribution of the bronchovascular bundles. Normal orientation of the pulmonary fissures. A few subcentimetric lymph nodes were noted, but there was no evidence of any pathological mediastinal or hilar nodal enlargement. The report concludes, apart from an enlarged parotid gland, an unremarkable CT of the neck and chest.

CT chest showed clear lungs and no nodules or masses. Normal pleural reflections with no collections, thickening, or masses. Multiple mediastinal lymph nodes. No detectable soft tissue or bony lesions in the chest wall.

Upper and lower endoscopy revealed severe gastritis with a transverse colon polyp; biopsies were NEGATIVE for high-grade dysplasia.

MRCP of the biliary system and the gall bladder was normal.

Liver biopsy shows three cores of liver tissue with adequate portal areas present for evaluation. The portal tracts harbor their usual structures. There is bile duct damage, “bile duct epithelial disorganization, prominent bile ductular proliferation, sinusoidal edema, and hepatocyte rosetting. Portal regions demonstrate moderate inflammatory infiltrates, comprising lymphocytes, plasma cells, and neutrophils with slightly increased eosinophils and moderate interface hepatitis. There are focal periportal hepatocytes swollen with the cytoplasm (cholestasis) and bile pigment accumulation within hepatocytes (bilirubinostasis). Bridging fibrosis is identified on trichrome stain.

Histopathological diagnosis

**Figure 3:**
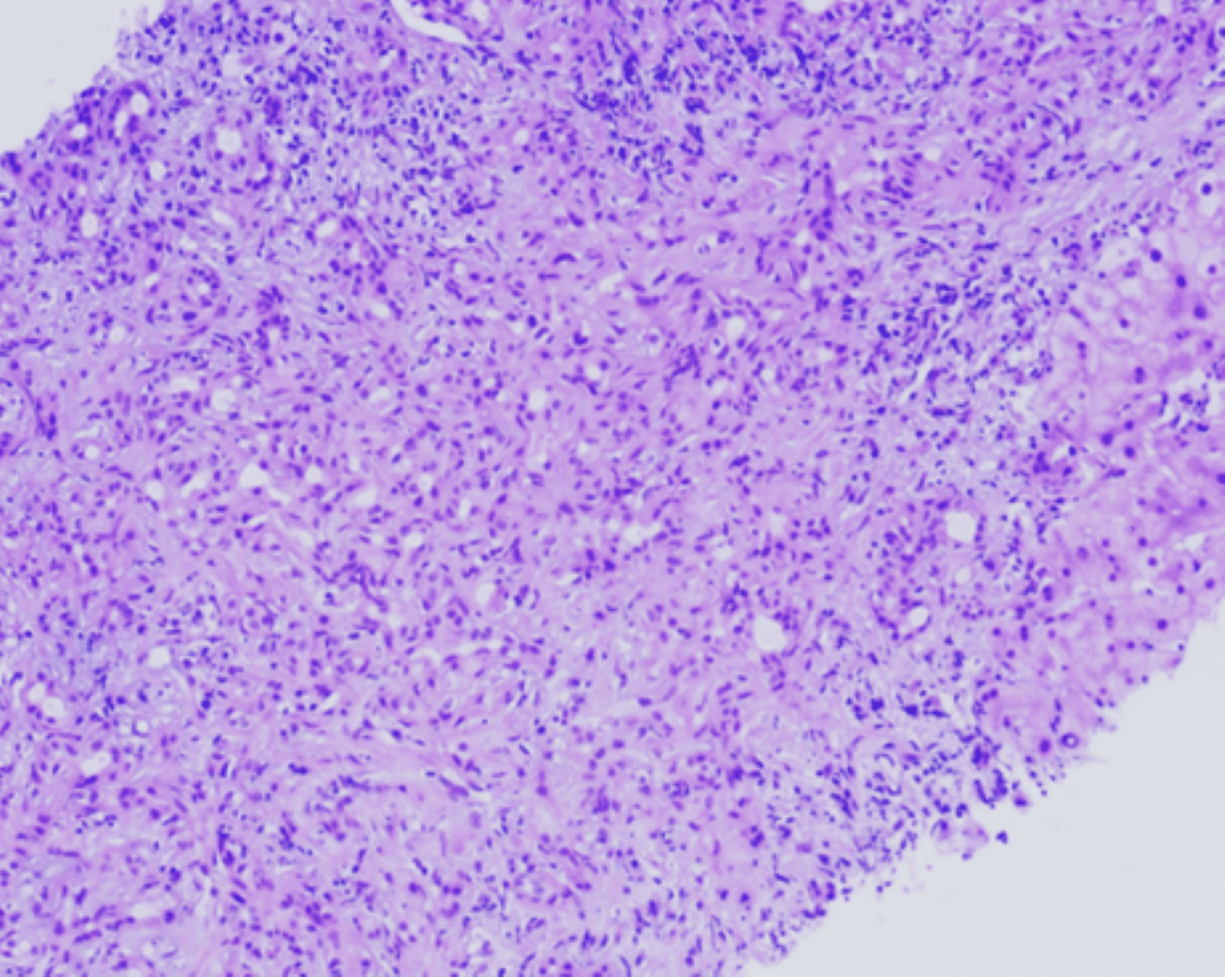
Liver biopsy showing a marked expansion of the portal tract by a storiform pattern of fibrosis surrounding exuberant bile ductular proliferation and lymphoplasmacytic infiltrate (hematoxylin-eosin, original magnification ×100).

**Figure 4:**
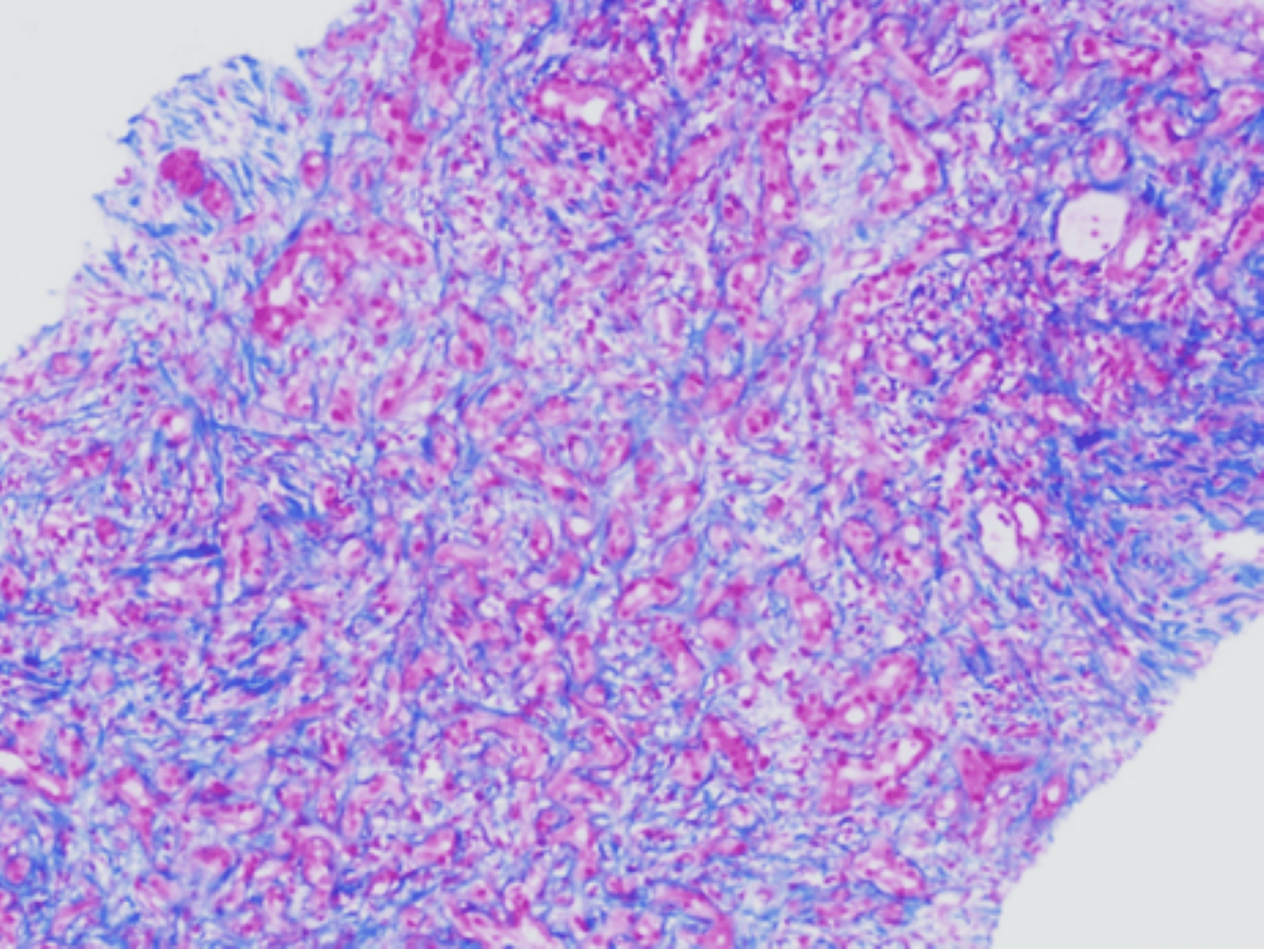
Portal tract fibrosis showing a storiform pattern highlighted with Masson trichrome staining, which highlights collagen (fibrosis) in blue (original magnification ×100).

**Figure 5:**
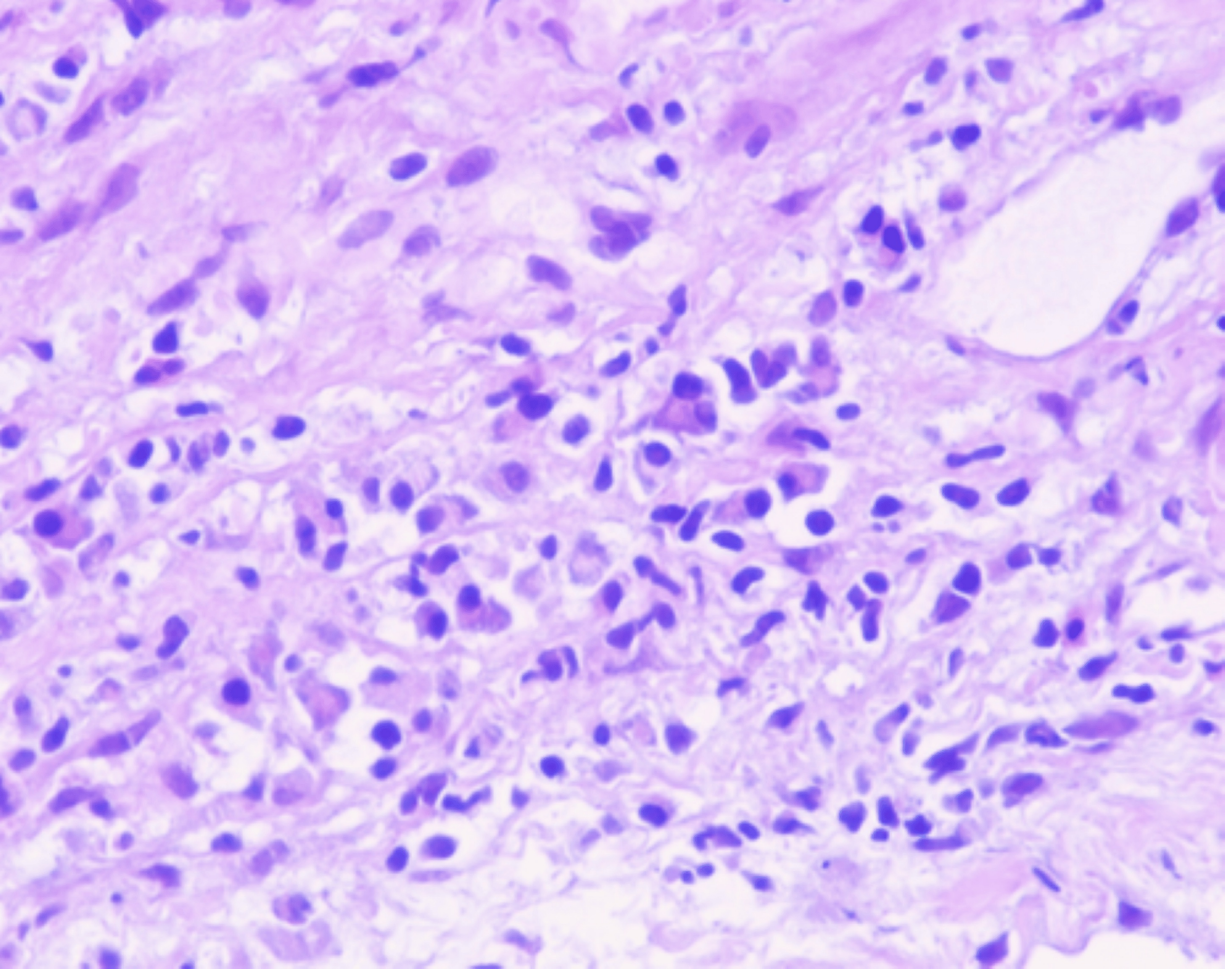
Plasma cell aggregates are mixed with small lymphocytes and a few eosinophils in the portal tract (hematoxylin-eosin, original magnification ×400).

**Figure 6:**
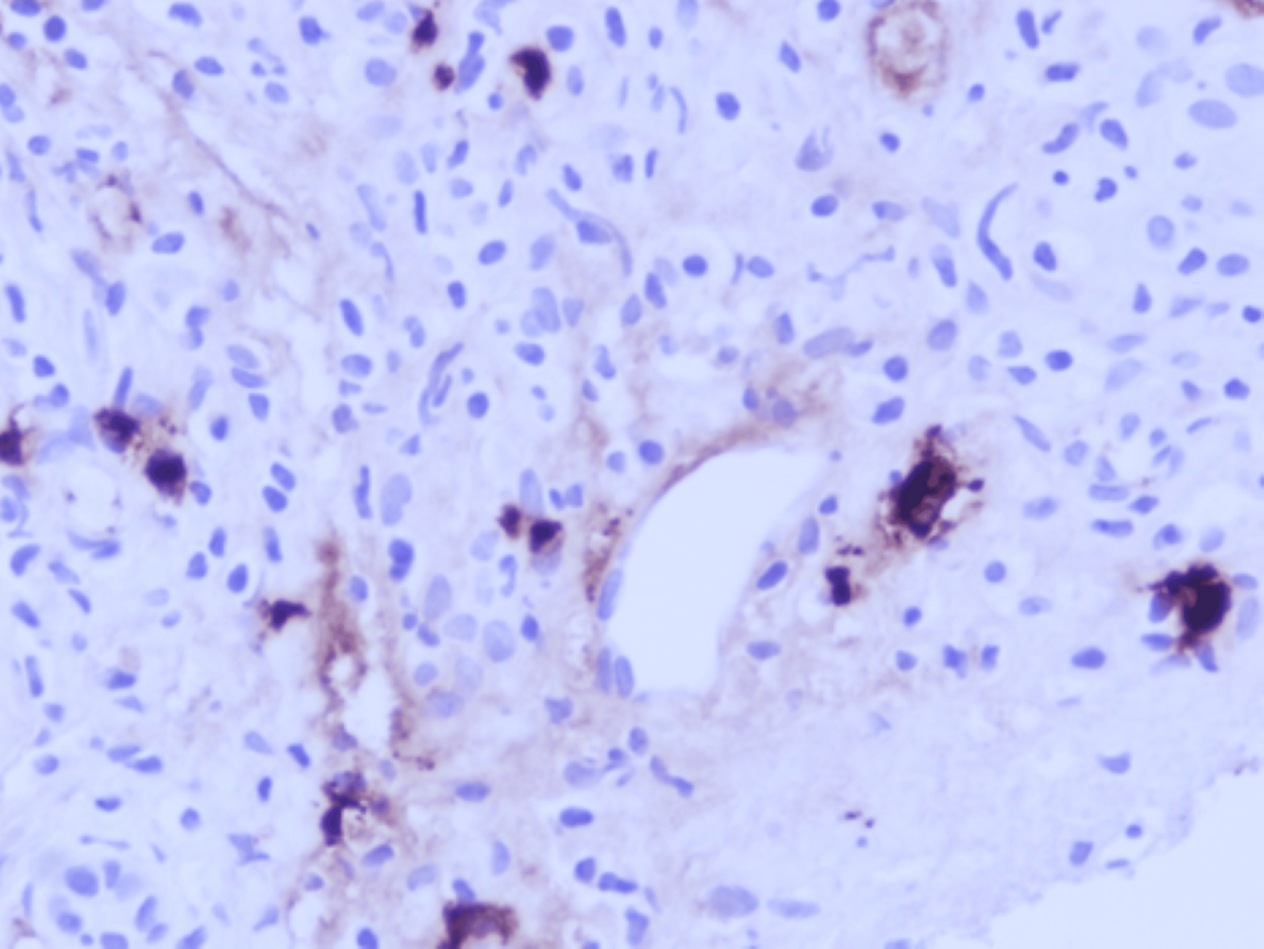
Immunohistochemical staining reveals numerous IgG4-positive plasma cells in the portal tract (original magnification ×400).

Chronic hepatitis of moderate activity with bridging fibrosis (Batts and Ludwig Grade 3 of 4, Stage 3 of 4). Microscopic examination of the liver biopsy revealed a portal-based, moderately dense lymphoplasmacytic infiltrate, admixed with neutrophils and scattered eosinophils. There was mild bile ductular proliferation and focal portal storiform fibrosis. No features of obliterative phlebitis were observed. These histological findings are illustrated in Figure 3, which shows portal tract expansion with storiform fibrosis and surrounding bile ductular proliferation, and in Figure 4, where the fibrosis pattern is highlighted using Masson trichrome staining. Due to the substantially raised serum IgG4 level, immunohistochemical staining of the IgG and IgG4 was performed. This illustrated 7-10 IgG4-positive plasma cells per high-power field and an IgG4/IgG plasma cell ratio of 50-60 percent, which was consistent with IgG4-related hepatobiliary disease. Plasma cell clusters with intermingled small lymphocytes and eosinophils are observed in Figure 5, and there are many IgG4-positive plasma cells in the portal tract in Figure 6. The patient met the 2020 Revised Comprehensive Diagnostic criteria of definite IgG4-related disease: the three domains of clinical, serological, and histopathological were met with the absence of obliterative phlebitis. The case also satisfied the 2016 ACR/EULAR classification criteria for primary Sjögren’s syndrome (SS) based on compatible clinical manifestations and serology.

Treatment and response

The patient was started on prednisolone 40 mg orally daily + hydroxychloroquine 200 mg orally twice daily + azathioprine 50 mg PO daily. The rheumatology team has decided that in cases of refractory disease to steroids and azathioprine treatment, they will be initiated on rituximab (lymphoma dose 375 mg/m²) weekly 4 times, and 1 g after 2 weeks, to be repeated every 6 months.

## Discussion

As mentioned earlier, IgG4-related disease (IgG4-RD), originally linked with autoimmune pancreatitis in 2001 [1], has subsequently been recognized as a multisystemic inflammatory condition that can affect various body regions. Although the underlying mechanisms remain unclear, dysregulated autoimmune reactions targeting possible local antigens or microbial agents are considered initiating factors, leading to a Th2-predominant immune response with increased cytokine production (IL-4, IL-5, and IL-13), promoting B cell class conversion and heightened IgG4 production [5]. Our diagnostic conclusion is additionally supported by the 2020 Revised Comprehensive Diagnostic (RCD) criteria for IgG4-RD, published by Umehara et al in Modern Rheumatology in 2021under Japanese Ministry of Health supervision [6]. These recommendations have been widely implemented for standardizing IgG4-RD identification across organ systems and encompass three categories: (1) clinical manifestations, including enlargement, mass formation, or nodular/hypertrophic abnormalities in an organ; (2) serological confirmation of increased serum IgG4 (>135 mg/dL); and (3) histopathological features, particularly dense lymphoplasmacytic infiltration with >10 IgG4+ plasma cells per high-power field and an IgG4+/IgG+ plasma cell proportion >40%, accompanied by storiform fibrosis and/or obliterative phlebitis. Our case satisfied all necessary criteria: hepatomegaly and bilateral parotid gland swelling (clinical category), significantly raised serum IgG4 at 4.31g/L (serological category), and liver biopsy revealing storiform fibrosis, 7-10 IgG4+plasma cells/HPF, and an IgG4+/IgG+ plasma cell proportion of 50-60%(histopathological category), thus fulfilling the requirements for definitive IgG4-RDaccording to RCD guidelines. Table 2 summarizes the reported cases and reviews describing IgG4-related autoimmune hepatitis (IgG4-AIH) and its overlap with autoimmune conditions such as SS, highlighting clinical features, histopathology, treatment, and outcomes.

**Table 2:** Comparison of the Current Case with Reported Literature on IgG4-Associated Autoimmune Hepatitis This table summarizes the clinical characteristics, serological profiles, histopathological findings, and therapeutic outcomes of the presented case alongside previously reported cases and systematic reviews of IgG4-related disease (IgG4-RD) and IgG4-associated autoimmune hepatitis (IgG4-AIH). It highlights the overlapping autoimmune associations, specifically with autoimmune pancreatitis and Sjögren’s syndrome.

Author (Year)	Patient Description	Associated Autoimmunity	Serum IgG4	Histology Findings	Treatment	Outcome
Our Case (2025)	56M, RUQ pain, jaundice, dry eyes/mouth	Primary Sjögren’s syndrome (anti-SSA+)	4.31 g/L (↑↑)	Interface hepatitis, rosettes, 7–10 IgG4+ plasma cells/HPF, IgG4: IgG ratio 50–60%	Prednisone + Azathioprine + HCQ; Rituximab planned	Improved liver function
[1]	Case with unexplained hepatitis, elevated ALT/ALP/γ‑GT, and IgG	Autoimmune pancreatitis	Markedly elevated (exact mg/dL unspecified)	Interface and lobular hepatitis, rosettes, abundant IgG4+ plasma cells in portal tracts	Glucocorticoids ± azathioprine	Good response; normalized liver tests
[4]	Adult male with elevated liver enzymes	None reported	Significantly elevated	Hepatitis with interface inflammation, IgG4+ plasma cell-rich infiltrates	Steroids	Complete remission
[7]	Case with AIP-associated IgG4‑AIH	Autoimmune pancreatitis	Elevated serum IgG4 (exact value unspecified)	Interface hepatitis, abundant IgG4 infiltrate	Steroid ± immunosuppressive therapy	Clinical resolution, sustained remission
[8]	Case-based review of GI IgG4-RD	AIH overlap	Variable (↑ or normal)	Interface hepatitis, portal inflammation, IgG4+ cells, storiform fibrosis	Steroids ± immunosuppressants	Often responsive
[9]	Cohort of IgG4-RD patients post-steroid therapy	Multi-organ IgG4-RD	High baseline IgG4	Not specified	Long-term low-dose steroids or immunosuppressants	40% relapse risk
[10]	Systemic review of IgG4-RD	Includes pSS overlaps	>135 mg/dL	IgG4: IgG >40%, storiform fibrosis	Prednisone, Rituximab	Relapse risk 30–40%

Macroscopically, this condition produces generalized organ enlargement and pseudo-tumorous mass development with distinctive lymphoplasmacytic infiltrates, elevated IgG4+ plasma cells, and storiform fibrosis. Despite advances, our understanding remains fragmented. To date, over 40 organs have been reported as potential targets of IgG4-RD. Among the most frequently involved are the salivary glands, particularly in IgG4-related sialadenitis (IgG4-RS) [10]. Sjogren syndrome (SS) is an autoimmune disease that mainly affects exocrine glands, especially salivary and lacrimal glands, and is manifested with typical symptoms of xerostomia and xerophthalmia. It is subdivided into primary SS (independent of other connective tissue diseases) and secondary SS (concurrent with other CTDs). Primary Sjogren syndrome (SS) is additionally categorized into glandular and extra-glandular variants. IgG4-RS and SS share clinical features such as glandular swelling, sicca symptoms, arthralgias, hypergammaglobulinemia, and ANA positivity. However, IgG4-RS is usually negative for anti-Ro/La antibodies, demonstrates abundant IgG4+ plasma cell infiltration in salivary tissue, and shows prompt steroid responsiveness. A comparison between SS and IgG4-related sialadenitis and dacryoadenitis reveals key clinical, immunological, and therapeutic distinctions that are critical for accurate diagnosis and treatment. While both conditions can present with salivary gland involvement and sicca symptoms, they differ notably in age of onset, gender predilection, immunoglobulin profiles, autoantibody presence, and response to corticosteroids. These differences are described in Table 3 and show the characteristic features of IgG4-related disease represented by the prevalence of IgG4-positive plasma cells, high serum IgG4 levels, and the significantly better response to glucocorticoid therapy. Understanding these differences is crucial for differentiating IgG4-RD from SS, especially in concurrent clinical presentations.

**Table 3:** Distinguishing Features Between Sjögren’s Syndrome and IgG4-Related Sialadenitis/Dacryoadenitis This table outlines the critical clinical, immunological, and histopathological differences between classic Sjögren’s syndrome (SS) and IgG4-related glandular disease. It emphasizes key discriminators, including age of onset, gender distribution, specific autoantibody profiles (anti-SS-A/anti-SS-B vs. IgG4), and the differential response to corticosteroid therapy.

Feature	Sjögren’s Syndrome (SS)	IgG4-Related Sialadenitis/Dacryoadenitis
Typical Age of Onset	40–50 years	50–60 years
Gender Distribution	Predominantly affects females	Nearly equal male-to-female ratio
Dry Eyes and Dry Mouth	Common	Rare or mild
Salivary/Lacrimal Gland Swelling	Often recurrent and spontaneously resolving, submandibular gland swelling is rare	Persistent, significant swelling often involves submandibular glands
Allergic Comorbidities	Rare	Common (e.g., allergic rhinitis, asthma)
Rheumatoid Factor / ANA	Frequently positive	Usually, negative
Anti-SS-A / Anti-SS-B Antibodies	Positive in ~70% (SS-A), ~30% (SS-B)	Typically, negative
Elevated Immunoglobulin Classes	IgG, IgA, IgM	IgG, IgE
IgG Subclasses Elevated	IgG1, IgG3	IgG4, IgG2
IgG4+ Plasma Cell Infiltration	Absent	Prominent
Lymphoepithelial Lesions	Frequently observed	Rare
Response to Corticosteroids	Generally poor	Rapid and effective
Recovery of Secretory Function	Limited improvement	Significant improvement

AIH is an autoimmune liver disease characterized by high serum IgG, interface hepatitis, lymphoplasmacytic infiltration, and rosette formations. SS may feature hepatic involvement, mainly via PBC or AIH. Distinguishing viral from autoimmune liver disease becomes crucial because treatment differs accordingly. Type 1 AIH is commonly seen in ANA, SMA, and p-ANCA and is highly responsive to steroid therapy. Conversely, type 2 AIH is rare but more severe; the pediatric and Mediterranean populations are primarily affected by it through anti-LKM antibodies. In primary SS, AIH occurs in 4-47% of patients, with Type 1 being the most common. Raised IgG4 levels occur in about 10% of patients with pSS, indicating a possible overlap or misclassification with IgG4-RD [11]. In such patients, particularly those with elevated liver enzymes, IgG4-RD should be considered, especially when accompanied by autoimmune pancreatitis or retroperitoneal fibrosis [5]. Only limited case reports of IgG4-positive plasma cell hepatic infiltration, specifically IgG4-AIH, have been documented. The initial case was reported in 2007 with increased ALT, ALP, γGTP, IgG, and IgG4 concentrations and interface plus lobular hepatitis. This specific case satisfied IAIHG criteria with substantial portal infiltration by IgG4+ plasma cells [1]. Another hepatic expression of IgG4-RD is the inflammatory pseudotumor. Two variants exist: fibro-histiocytic and lymphoplasmacytic. The latter is defined by the massive IgG4+ plasma cell infiltration and obliterative phlebitis and is considered a liver manifestation of IgG4-RD. Glucocorticoids represent the primary therapeutic agents in treating active IgG4-AIH. Remission is typically achieved with oral prednisone (0.6-0.8 mg/kg/day for 4 weeks), subsequently tapered based on clinical and biochemical improvement. To prevent relapse, maintenance treatment with low-dose prednisone (2.5-5 mg/day) can be established. For extended maintenance and avoiding steroid dependency, azathioprine is favored. Initial doses are 50 mg/day, and the dosage is increased to 1-2 mg/kg/day while monitoring carefully. Rituximab, a B-cell depleting medication, is recommended for inpatients with recurring or resistant disease [9]. This developing clinical condition again necessitates definitive diagnostic criteria as well as therapeutic protocols, especially when IgG4-RD coexists with classical autoimmune disorders.

## Conclusions

The case describes a clinically significant co-occurrence of IgG4-related autoimmune hepatitis (IgG4-AIH) and primary Sjogren syndrome (pSS) that has occurred infrequently. High serum levels of IgG4, storiform fibrosis, and widespread IgG4+ plasma cell infiltration established this diagnosis of IgG4-AIH. There was subsequently a concurrent presence of pSS, demonstrated by the detection of anti-Ro antibodies and sicca symptoms. Approximately one in ten patients with pSS has high IgG4 levels, which may represent a different subset of the disease or incorrectly diagnosed patients with IgG4-related disease (IgG4-RD). Such a grey area in diagnosis would warrant a greater suspicion and histopathological evaluation in cases of unexplained liver dysfunction in pSS. Management with corticosteroids and azathioprine yielded clinical improvement in this patient, and rituximab was initiated as a biologic option to prevent relapse. However, due to the rarity of IgG4-AIH, there are currently no universally accepted diagnostic or treatment guidelines, and evidence is limited to case reports and small series. Furthermore, the case adds insight into the clinical profile, diagnosis, and response to treatment, and therefore underlines the importance of standardized criteria to characterize IgG4-AIH as a variant of classical AIH or a hepatic variant of systemic IgG4-RD. Multicenter and longitudinal studies should hence be carried out to better describe this entity and better dictate evidence-based clinical practice.
